# Comparison of Treatment Effect Estimates for Pharmacological Randomized Controlled Trials Enrolling Older Adults Only and Those including Adults: A Meta-Epidemiological Study

**DOI:** 10.1371/journal.pone.0063677

**Published:** 2013-05-28

**Authors:** Valérie Seegers, Ludovic Trinquart, Isabelle Boutron, Philippe Ravaud

**Affiliations:** 1 Assistance Publique-Hôpitaux de Paris, Hôpital Hôtel-Dieu, Paris, France; 2 INSERM U738, Paris, France; 3 Université Paris Descartes – Sorbonne Paris Cité, Paris, France; 4 French Cochrane Centre, Paris, France; 5 Department of Epidemiology, Columbia University Mailman School of Public Health, New York, New York, United States of America; University Medical Center Rotterdam, The Netherlands

## Abstract

**Context:**

Older adults are underrepresented in clinical research. To assess therapeutic efficacy in older patients, some randomized controlled trials (RCTs) include older adults only.

**Objective:**

To compare treatment effects between RCTs including older adults only (elderly RCTs) and RCTs including all adults (adult RCTs) by a meta-epidemiological approach.

**Methods:**

All systematic reviews published in the Cochrane Library (Issue 4, 2011) were screened. Eligible studies were meta-analyses of binary outcomes of pharmacologic treatment including at least one elderly RCT and at least one adult RCT. For each meta-analysis, we compared summary odds ratios for elderly RCTs and adult RCTs by calculating a ratio of odds ratios (ROR). A summary ROR was estimated across all meta-analyses.

**Results:**

We selected 55 meta-analyses including 524 RCTs (17% elderly RCTs). The treatment effects differed beyond that expected by chance for 7 (13%) meta-analyses, showing more favourable treatment effects in elderly RCTs in 5 cases and in adult RCTs in 2 cases. The summary ROR was 0.91 (95% CI, 0.77–1.08, p = 0.28), with substantial heterogeneity (I^2^ = 51% and τ^2^ = 0.14). Sensitivity and subgroup analyses by type-of-age RCT (elderly RCTs vs RCTs excluding older adults and vs RCTs of mixed-age adults), type of outcome (mortality or other) and type of comparator (placebo or active drug) yielded similar results.

**Conclusions:**

The efficacy of pharmacologic treatments did not significantly differ, on average, between RCTs including older adults only and RCTs of all adults. However, clinically important discrepancies may occur and should be considered when generalizing evidence from all adults to older adults.

## Introduction

The number of people aged 60 years and older now is twice the number in 1980. By 2050, the number of people 80 years and older will be four-fold the current number [1]. The life expectancy has increased during the last decades and thus, more older people may need medical interventions.

The care of older patients presents a challenge for evidence-based medicine [2]: the body of evidence concerning the efficacy of therapeutic interventions in older adults is usually small because these patients are largely underrepresented in clinical trials [3], [4], [5], [6], [7], [8], [9]. Nearly three-quarters of trials reported in major journals excluded older patients [10]. This exclusion is often poorly justified despite the availability of methods for enhancing their recruitment [11], [12], [13], [14] and several authorities highlighting the need for representative samples of older adults in randomized controlled trials (RCTs) [2], [15], [16], [17] and even the need for randomized trials including older adults only [18], [19].

In the absence of evidence for older adults, pharmacologic results for younger adults are commonly extrapolated to older patients assuming that the efficacy is the same. However, this practice may not be valid because of physiological changes associated with aging that affect the pharmacokinetics of drugs, including multiple co-morbidities in older people and unpredictable treatment responses [20], [21]. Indeed, the Randomized Aldactone Evaluation Study [22] demonstrated reduced mortality with spironolactone administration in patients with congestive heart failure, with average age 60 years. In contrast, data from several observational cohorts of patients with congestive heart failure who were 13 years older revealed that spironolactone was associated with an almost four-fold increase in number of deaths due to hyperkaliemia [23]. We need to be able to draw on the results of good-quality research to inform best practice in the management of older adults.

In this study, we assessed empirically whether treatment effects differed between RCTs including older adults only (elderly RCTs) and RCTs including all adults (adult RCTs) that were performed for the same medical problem and with the same pharmacological therapeutic interventions, using a meta-epidemiological approach [24].

## Materials and Methods

We performed a meta-epidemiological study to compare treatment effect estimates between elderly RCTs and adult RCTs [25]. We identified a large number of meta-analyses that included at least one elderly RCT and at least one adult RCT and concerned a variety of conditions and therapeutic interventions. We recorded whether individual RCTs included older adults only or not. For each meta-analysis, we compared summary odds ratios (ORs) for elderly RCTs and adult RCTs by calculating a ratio of ORs (ROR). A summary ROR was estimated across all meta-analyses.

This methodology has been used previously to establish empirical evidence of bias in estimating treatment effects (e.g., double blinding, intent-to-treat analyses and allocation concealment [26], [27], [28], [29], single vs multi-center [30], [31], randomized vs non-randomized trials [32]) and recently to compare efficacy in trials of children and adults [33].

### Selection of meta-analyses

One reviewer screened all systematic reviews published in the Cochrane Library in the Cochrane Database of Systematic Reviews (Issue 4, 2011) concerning all medical domains assessing pharmacological therapeutic interventions. Reviews for Cochrane Groups working on pregnancy, neonatality, fertility, cancer childhood, organization care and public health were excluded.

Eligible reviews reported at least one meta-analysis of a binary outcome for at least 3 RCTs, of which 1 was specific to older adults (elderly RCT) and 1 not specific to older adults (adult RCT). We focused on Cochrane reviews because some studies have shown that these have high methodological quality and are well reported [34].

One reviewer selected reviews in 3 steps: examining the title, abstract and full-text for potentially relevant reviews. For each eligible systematic review, we systematically screened the “Characteristics of included studies” table to identify elderly RCTs as follows: the authors clearly reported the population as “older”, “geriatric” or “elderly” or they defined the lower age limit as selection criteria or the minimum age of included patients as ≥60 years. If the previous information was not available, we also considered trials with mean patient age ≥75 years as elderly RCTs. For adult RCTs, we distinguished 4 categories: trials excluding older adults (defined as RCTs excluding older adults), trials including both older adults and non-older adults (RCTs of mixed-age adults), unclear or unspecified age recruitment and children-specific trials.

### Data extraction

For each eligible meta-analysis, we extracted the following information from each Cochrane review: clinical domain, experimental and control interventions (placebo, no treatment, active drug), primary binary efficacy outcome, and number of included RCTs.

For each RCT, we extracted the age category reported in Table 1 of the Cochrane review, as described: elderly RCT or adult RCT (including RCTs excluding older adults, RCTs of mixed-age adults, unclear RCTs and children-specific RCTs). We also extracted the sample size and the number of patients and events per each study arm from the forest plot of the meta-analysis.

If more than 1 primary outcome was reported or if the primary outcome was not specified among several binary outcomes, we selected the first outcome presented in the Results section. When 2 active interventions were compared, identification of the experimental and control interventions was based on interpretation by the authors of the Cochrane review. If this identification was not clear, we identified which intervention was first discovered according to PubMed first indexing, and this was considered the control intervention. One of the authors extracted the data.

### Data synthesis and analysis

We used a two-stage model to compare treatment effect estimates for elderly RCTs and adult RCTs.

### Treatment effect estimates for elderly RCTs and adult RCTs

For each included meta-analysis, we estimated the 2 summary odds ratios (ORs) comparing the experimental and the control interventions for elderly RCTs and adult RCTs separately. In cases of many trials for an age group, we combined the trials by random-effects models [35]. Heterogeneity across RCTs was assessed by means of the I^2^ statistic and the between-RCT variance τ^2^. Outcome events were re-coded so that an OR <1 indicated a beneficial effect of the experimental intervention. When appropriate, we used a continuity correction [36].

For each meta-analysis, we evaluated whether the difference in ORs between elderly RCTs and adult RCTs was larger than would be expected by chance alone using z scores. We also assessed in how many cases the summary OR for elderly RCTs was less than half the summary OR for adult RCTs and in how many cases the summary OR for elderly RCTs was twice the summary OR for adult RCTs.

### Meta-analysis of ratios of odds ratios between elderly RCTs and adult RCTs

For each meta-analysis, we estimated an ROR and associated standard error [37] from the summary ORs estimated for elderly RCTs and adult RCTs. A ROR<1 indicated that elderly RCTs yielded larger estimates of the intervention effect than did adult RCTs. Then we combined all RORs across meta-analyses by using a random-effects meta-analysis model [38]. Heterogeneity across meta-analyses was assessed by means of the I^2^ statistic and the between–meta-analyses variance τ^2^. We also computed a prediction interval for the summary ROR [39].

### Age-group RCT analyses

In our main analysis, we compared elderly RCTs and adult RCTs. In a complementary analysis, we compared elderly RCTs to RCTs excluding older adults and to RCTs of mixed-age adults. Unclear RCTs were alternatively considered as RCTs excluding older adults or as RCTs of mixed-age adults.

### Exploration of heterogeneity

We used a graphical method to identify meta-analyses that contributed considerably to the overall heterogeneity across RORs and that could strongly influence the overall summary ROR [40]. In a sensitivity analysis, we excluded the most heterogeneous and influential meta-analyses and re-estimated the summary ROR and the heterogeneity statistics across meta-analyses. To further explore heterogeneity, we performed 2 pre-specified subgroup analyses by type of comparator treatment (active drug vs placebo and/or usual care or no treatment) and type of outcome (mortality vs other outcomes).

### Statistical analysis

There is no validated method of power calculation for meta-epidemiological analyses. Statistical power of a meta-epidemiological analysis would depend on the true ROR, the number of meta-analyses and trials, the distribution in each meta-analysis of trials with and without the characteristic of interest, and the heterogeneity between and within studies. We arbitrarily prespecified a sample size of 50 meta-analyses.

The analyses involved the two-stage comparison described above and were consolidated with a one-stage multilevel model (ie, a hierarchical logistic regression model with mixed effects) [41]. We used R v2.15.1 [42] for all analyses and the packages meta [43], metafor [44] and lme4 [45].

## Results

Of the 3, 403 systematic reviews identified from the Cochrane Library, 55 were included (**Figure S1**). Their characteristics are in **Table S1**. Fifteen (27%) concerned psychiatry, 14 (25%) cardiovascular diseases and 9 (16%) neurology. In total, 35 (64%) meta-analyses compared pharmacologic treatment with placebo or no treatment. The 55 meta-analyses involved 524 RCTs overall (**Figure S1**): 90 (17%) were elderly RCTs, and 434 were adult RCTs, including 73 RCTs excluding older adults, 224 RCTs of mixed-age adults, 133 unclear RCTs and 4 children-only RCTs. The median sample size was 91 patients (min-max 18–6,706) for elderly RCTs and 82 (10–23,323) for adult RCTs. The total number of included patients was 199,760, with 21% for elderly RCTs and 79% for adult RCTs.

### Treatment effect estimates for elderly RCTs and adult RCTs

Among the 55 meta-analyses, for 48, the summary ORs did not differ between elderly RCTs and adult RCTs. However, the summary ORs differed beyond chance for 7 (13%) meta-analyses (**Figure S2**), showing more favourable treatment effects in elderly RCTs for 5 meta-analyses (CD000096, CD00424, CD001886, CD003781, CD008120) and in adult RCTs for 2 meta-analyses (CD002747, CD007503). The characteristics of the 7 corresponding reviews are in **Table S2**.

When we considered the magnitude of treatment effect estimates, the summary OR for elderly RCTs was less than half the summary OR for adult RCTs for 9 (16%) meta-analyses. In contrast, the summary OR for elderly RCTs was at least twice the summary OR for adult RCTs for 8 (15%) meta-analyses.

### Meta-analysis of RORs between elderly RCTs and adult RCTs

The results of the meta-analysis of RORs are in **Figure S3**. The summary ROR across all 55 included meta-analyses was 0.91 (95% CI, 0.77–1.08, p = 0.278), with substantial heterogeneity (I^2^ = 51.1% and τ^2^ = 0.137). The 95% prediction interval, which provides a predicted range for the true ROR in an individual meta-analysis, was 0.43 to 1.92.

### Age-group RCT analyses

In comparing elderly RCTs with RCTs excluding older adults and RCTs of mixed-age adults, the summary ROR was 0.88 (95% CI 0.63–1.17, n = 29 reviews) and 0.93 (0.76–1.15, n = 38 reviews), respectively. The hierarchical multilevel modeling approach yielded similar summary RORs (elderly RCTs vs RCTs excluding older adults, 0.88, 0.70–1.09; elderly RCTs vs RCTs of mixed-age adults, 0.95, 0.83–1.09). Alternatively, when we considered trials for which the age category was unclear as RCTs excluding older adults or RCTs of mixed-age adults, we did not find any difference (**Appendix S1**).

### Exploration of heterogeneity

Three meta-analyses (CD008120, CD003781, and CD002747) accounted for most of the heterogeneity (50.0%) and had a strong influence on the sROR (65% of the sum of the influences; Figure in **Appendix S1**). In a sensitivity analysis, the exclusion of these 3 meta-analyses strongly reduced the heterogeneity across RORs (I^2^ = 2%; τ^2^ = 0.0035), but the summary ROR was only slightly modified (0.99, 95% CI 0.90–1.10). The characteristics of these reviews are in **Table S2**.

Subgroup analysis by type of control group revealed no statistically significant difference, on average, in treatment effects estimates between elderly RCTs and adult RCTs with control groups of placebo, no treatment or usual care (summary ROR 0.87; 95% CI 0.68–1.11) or an active drug (0.95; 0.82–1.10). Subgroup analysis by type of outcome yielded similar results when assessing mortality (1.11; 0.90–1.36) and other outcomes (0.95; 0.82–1.10).

## Discussion

For a sample of 55 Cochrane systematic reviews, we performed an empirical evaluation of the relative treatment effect estimates of pharmacological therapeutic interventions between RCTs that included older adults only and RCTs of the general adult population. In 7 cases, treatment effect estimates differed significantly between elderly RCTs and adult RCTs, showing more favourable treatment effects in elderly RCTs for 5 meta-analyses and in adult RCTs for 2 meta-analyses. Treatment effects estimates did not differ significantly, on average, between elderly RCTs and adult RCTs.

For some elderly RCTs, the intervention efficacy would have been overestimated if based on adult RCTs (meta-analyses CD002747, CD007503). In these 2 meta-analyses, the experimental treatment may have suggested benefit in the global population, but for RCTs specific to older adults, the control treatment may have been more beneficial than the experimental treatment. The first identified situation concerned women with metastatic breast cancer. For adult RCTs, the results of the meta-analysis would have leaned toward chemotherapy alone, whereas for older women, the results would have favored endocrine therapy alone. This situation is unique in terms of menopausal status, which depends on the age of women. The second situation involved psychiatry (antidepressants for depression in physically ill people). However, in this meta-analysis, one elderly RCT involved patients in a general medical ward for older adults, whereas another included RCTs of mostly outpatients with chronic diseases (chronic prostatitis, Parkinson disease, multiple sclerosis and cancer). The type of depression could differ among these types of illnesses.

The lack of evidence from elderly RCTs may have 2 consequences: the use of medications with risks that are likely to out-weight benefits and undertreatment of older patients, which may result in a lack of improvement [46], [47], [48], [49]. In practice, the allocation of a pharmacological treatment for an older patient is guided by determination of the absolute benefits and harm in individual patients. Unfortunately, many reports of RCTs fail to provide detailed adverse effects data, and the quality of those that do is poor [50], [51], [52], [53].

The definition of older patients is controversial. For example, the European Medicines Agency arbitrarily defines its scope as patients 65 years of age (standard retirement age) or older [16], the World Health Organization proposed a cut-off of 60 years or older [1], and several medical domains have a cut-off of 80 years and older [54]. At the present and hopefully in the next decades, people in their 60 s or even 70 s will become healthier. Nevertheless, because of the physiological changes of aging (eg, weight, body composition, decreased glomerular filtration rate) and the increased risk of neurodegenerative diseases, diabetes, and cardiovascular events, for example, this age group differs from younger adults. Although aging is a progressive process, the definition of older adults in RCTs should be based on available and relevant indicators.

Our study contains some limitations. First, for consistency, we considered only reviews with binary efficacy outcomes only; however, this involves a large portion of clinically useful outcomes in clinical trials. Second, some Cochrane reviews did not provide information on the age of trial populations and, in theory, some of these meta-analyses might have been eligible if they included both elderly RCTs and adult RCTs. However, screening thousands of trials with unspecified age distribution would have been difficult, with uncertain yield; therefore, we depended on the information collected and recorded by the Cochrane authors of reviews to decide whether to include the review. The quality of Cochrane reviews is generally considered very good [55]. Third, in our main analyses, we included RCTs with “unspecified/unclear” age group from each eligible meta-analysis, which accounted for 25% of the trials, but excluding or reclassifying them in sensitivity analyses with adult RCTs yielded similar results. Fourth, the lack of data did not allow for performing formal comparisons of different older age groups (eg, older vs very older). Fifth, the results of both elderly RCTs and adult RCTs may be biased for diverse reasons, and the meta-analysis results may also be affected. Some reviews perform quality and bias assessments, but these assessments can differ across reviews, [56] and associations of reported quality with treatment effects may be tenuous [57]. However, biases are unlikely to differ in elderly RCTs and adult RCTs.

Our findings reflect only efficacy of treatment as measured in randomized trials. This efficacy may differ from effectiveness in clinical practice because patients included in trials may differ from patients in clinical practice. Some meta-confounding factors could bias our findings. These factors may be related to differences between included trials (concerning patient inclusion critera, the risk of bias of trials). Elderly patients included in elderly RCTs may be over-selected as compared with other adults included in general-age adult trials because of inclusion criteria in RCTs (eg, comorbities, illness severity, physiological functions, in- and outpatients). Taking such biases into account in the analyses may be difficult, but identifying such confounders may be useful. Therefore, we checked the co-morbidities used as selection criteria of trials included in meta-analyses. No trial had been excluded because of co-morbidities or physical or biological age-related dysfunctions. Nevertheless, we did not check patient exclusion criteria for individual elderly RCTs. Our exploratory framework does not allow for concluding that pharmacological treatment effects in elderly patients are similar to those in non-elderly patients because we explored trials that included only elderly patients as compared with trials that included adults without lower-age–inclusion criteria. Similar results of treatment effect estimates for pharmacologic RCTs including elderly patients only and adult RCTs were average estimates, but discrepancies in treatment effects can occur. The extent and direction of the difference were unpredictable, and extrapolation of evidence from adult RCTs to elderly RCTs can sometimes be tenuous.

As Scott and Guyatt state, [2] “*It is wisest to assume similar relative treatment effects in older and younger patients unless there is compelling evidence of age-related differences.* ^”^ Identification of interventions that do not provide any clinical benefit in older versus younger adults or are potentially harmful in older adults is important. Similarly, current European regulations require mandatory studies of older adults for all drugs,^15^ and the European Medicines Agency plans to make publicly available all results of clinical trials of older adults, but this move does not guarantee that sufficiently large trials will be conducted. Generation of large-scale evidence for geriatric indications is warranted.

## Supporting Information

Figure S1 **Flow-chart of included reviews.**
(EPS)

Figure S2 **Comparison of the summary odds ratios (ORs; and 95% confidence intervals [95% CIs]) for RCTs specifically including and not including older adults (elderly RCTs and adult RCTs).** Summary ORs were estimated with random-effects meta-analysis. Data in blue indicate meta-analyses for which the difference between summary ORs in elderly RCTs and in adult RCTs was beyond what would be expected by chance alone. Labels in bold indicate meta-analyses for which the magnitude of the treatment effect estimates was ≥2 or ≤50%. **OR<1 favors experimental treatment and OR >1 favors the control treatment.** *One meta-analysis showed the experimental intervention to be significantly worse than the control (CD003348 “Patient controlled opioid analgesia versus conventional opioid analgesia for postoperative pain”).
(TIFF)

Figure S3 **Meta-analysis of ratios of ORs for elderly RCTs and adult RCTs.** The width of the diamond is the 95% confidence interval for the true summary ROR and the dotted line is the prediction interval which indicates the possible ROR in an individual meta-analysis.
(TIFF)

Table S1 **Description of meta-analyses including at least 1 elderly randomized controlled trial (RCT specific to older adults) and including at least 1 adult RCT (not specific to older adults).**
(DOC)

Table S2 **Characteristics of meta-analyses with significantly different treatment effect estimates between elderly RCTs and adult RCTs.** OR: odds ratio; 95% CI: 95% confidence interval, * the 3 reviews that accounted for most of the overall heterogeneity in ratio of ORs across all reviews.
(DOC)

Appendix S1 Annexes.
(DOCX)
