# Supplementary figures and images for: Comparison of Treatment Effect Estimates for Pharmacological Randomized Controlled Trials Enrolling Older Adults Only and Those including Adults: A Meta-Epidemiological Study
Source: PLoS One. 2013 May 28;8(5):e63677. doi: 10.1371/journal.pone.0063677 (PMC3665786; doi:10.1371/journal.pone.0063677)

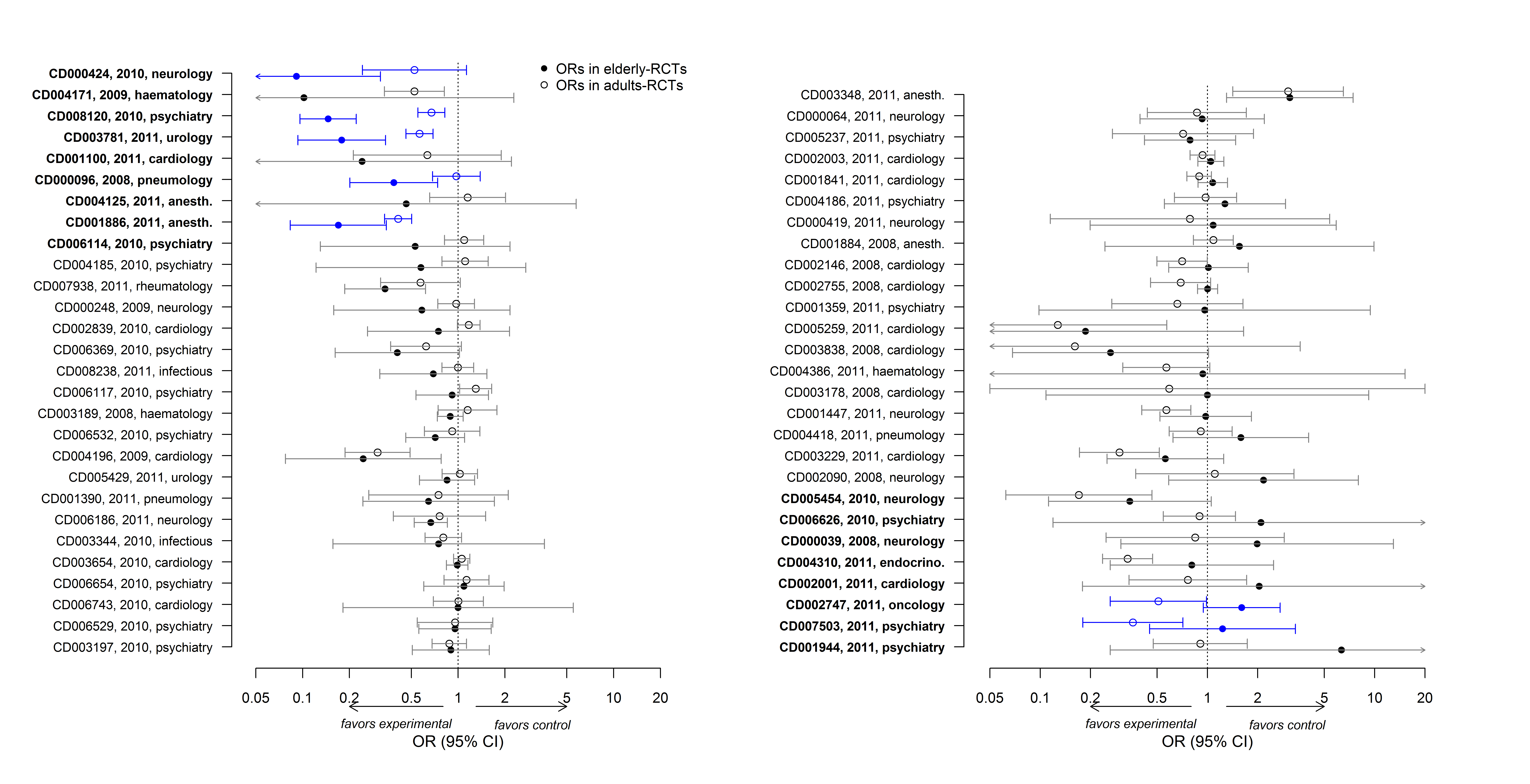

Supplement: Figure S2 — Comparison of the summary odds ratios (ORs; and 95% confidence intervals [95% CIs]) for RCTs specifically including and not including older adults (elderly RCTs and adult RCTs). Summary ORs were estimated with random-effects meta-analysis. Data in blue indicate meta-analyses for which the difference between summary ORs in elderly RCTs and in adult RCTs was beyond what would be expected by chance alone. Labels in bold indicate meta-analyses for which the magnitude of the treatment effect estimates was ≥2 or ≤50%. OR<1 favors experimental treatment and OR >1 favors the control treatment. *One meta-analysis showed the experimental intervention to be significantly worse than the control (CD003348 “Patient controlled opioid analgesia versus conventional opioid analgesia for postoperative pain”). (TIFF) [file pone.0063677.s002.tiff]

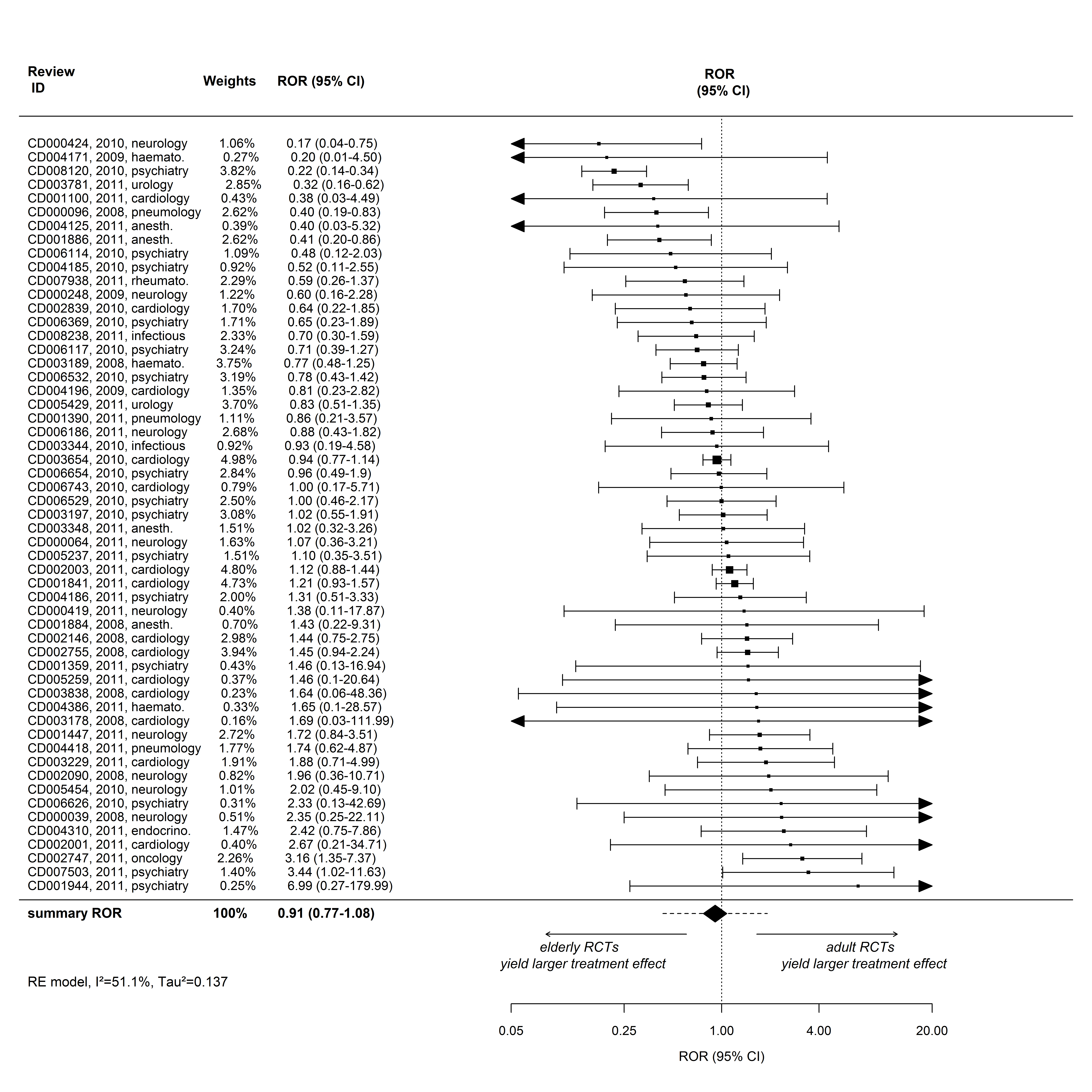

Supplement: Figure S3 — Meta-analysis of ratios of ORs for elderly RCTs and adult RCTs. The width of the diamond is the 95% confidence interval for the true summary ROR and the dotted line is the prediction interval which indicates the possible ROR in an individual meta-analysis. (TIFF) [file pone.0063677.s003.tiff]
