# Supplementary material for: Comparison of Treatment Effect Estimates for Pharmacological Randomized Controlled Trials Enrolling Older Adults Only and Those including Adults: A Meta-Epidemiological Study
Source: PLoS One. 2013 May 28;8(5):e63677. doi: 10.1371/journal.pone.0063677 (PMC3665786; doi:10.1371/journal.pone.0063677)
Supplement: Table S1 — Description of meta-analyses including at least 1 elderly randomized controlled trial (RCT specific to older adults) and including at least 1 adult RCT (not specific to older adults). (DOC) [file pone.0063677.s004.doc]

|  | **Meta-analyses (n=55)** |
| --- | --- |
| No. of RCTs included in the meta-analysis, median (min-max) | 7 (3-64) |
| Percentage of elderly RCTs in the meta-analysis, median % (min-max) | 20 (4.5-75) |
| Weight of elderly RCTs in the meta-analysis, median % (min-max) | 18.9 (2-86) |
| **Clinical domains** |  |
| Psychiatry | 15 (27%) |
| Cardiovascular | 14 (25%) |
| Neurology | 9 (16%) |
| Anesthesiology | 4 (7%) |
| Haematology | 3 (5%) |
| Pneumology | 3 (5%) |
| Infectious diseases | 2 (4%) |
| Urology | 2 (4%) |
| Endocrinology | 1 (2%) |
| Oncology (breast cancer) | 1 (2%) |
| Rheumatology | 1 (2%) |
| **Comparators** |  |
| Placebo or usual care | 35 (64%) |
| Active drug | 20 (36%) |
| **Outcome studied** |  |
| Mortality | 19 (35%) |
| Other | 36 (65%) |
