# Supplementary material for: Comparison of Treatment Effect Estimates for Pharmacological Randomized Controlled Trials Enrolling Older Adults Only and Those including Adults: A Meta-Epidemiological Study
Source: PLoS One. 2013 May 28;8(5):e63677. doi: 10.1371/journal.pone.0063677 (PMC3665786; doi:10.1371/journal.pone.0063677)
Supplement: Table S2 — Characteristics of meta-analyses with significantly different treatment effect estimates between elderly RCTs and adult RCTs. OR: odds ratio; 95% CI: 95% confidence interval, * the 3 reviews that accounted for most of the overall heterogeneity in ratio of ORs across all reviews. (DOC) [file pone.0063677.s005.doc]

| **ID** | **- Medical domain**  **- Experimental vs control treatment**  **- Outcome studied** | **No. of RCTs in meta-analysis** | **Overall meta-analysis: OR (95% CI); I²**  **Elderly RCTs: OR (95% CI); I²**  **Adult RCTs: OR (95% CI); I²** |
| --- | --- | --- | --- |
| CD000096 | -Acute stroke | 10 RCTs | Overall OR=0.81 (0.56-1.16); I²=20% |
|  | -IV Glycerol vs. avoided glycerol | 2 elderly RCTs | OR elderly RCTs=0.39 (0.20-0.74); I²=0% |
|  | -Death during the scheduled period | 8 adult RCTs | OR adult RCTs= 0.97 (0.68-1.39); I²=0% |
| CD000424 | - Treatment for aphasia following stroke | 4 RCTs | Overall OR=0.27 (0.08-0.88); I²=71% |
|  | - Piracetam vs placebo | 1 elderly RCT | OR elderly RCTs= 0.09 (0.03-0.32) |
|  | - No. of patients with aphasia not improved at the end of the study | 3 adult RCTs | OR adult RCTs=0.53 (0.24-1.13); I²=24% |
| CD001886 | - Anti-fibrinolytic use for minimising perioperative allogeneic blood transfusion | 64 RCTs | Overall OR=0.39 (0.32- 0.47); I²=41% |
|  | - Tranexamic acid vs. placebo or nothing | 5 elderly RCTs | OR elderly RCTs= 0.17 (0.08-0.35); I²=5% |
|  | - No. of patients exposed to allogenic blood | 59 adult RCTs | OR adult RCTs= 0.31 (0.34-0.50); I²=39% |
| CD002747* | - Metastatic breast cancer | 7 RCTs | Overall OR=0.79 (0.39-1.62); I²=75% |
|  | - Chemotherapy alone vs endocrine therapy alone | 2 elderly RCTs | OR elderly RCTs= 1.60 (0.95-2.72); I²=0% |
|  | - Tumor response rate | 5 adult RCTs | OR adult RCTs=0.51 (0.26-0.99); I²=52% |
| CD003781* | - overactive bladder syndrome | 8 RCTs | Overall OR=0.47 (0.34-0.65); I²=61% |
|  | - Anticholonergics vs. placebo | 3 elderly RCTs | OR elderly RCTs= 0.18 (0.09-0.34); I²=0% |
|  | - Patient perception of cure improvement | 5 adult RCTs | OR adult RCTs=0.56 (0.46-0.69); I²=17% |
| CD007503 | - Depression in physically ill people | 7 RCTs | Overall OR=0.44 (0.22-0.86); I²=60% |
|  | - Antidepressants vs placebo | 1 elderly RCT | OR elderly RCTs= 1.23 (0.45-3.36) |
|  | - Response to treatment (4-5w) | 6 adult RCTs | OR adult RCTs=0.36 (0.18-0.71); I²=52% |
| CD008120* | - Anxiety disorders | 4 RCTs | Overall OR=0.45 (0.22-0.91); I²=93% |
|  | - Quetiapine monotherapy vs placebo | 1 elderly RCTs | OR elderly RCTs= 0.15 (0.10-0.22) |
|  | - Response as defined by original study | 3 adult RCTs | OR adult RCTs=0.67 (0.55-0.82); I²=0% |
