# Supplementary material for: Comparison of Treatment Effect Estimates for Pharmacological Randomized Controlled Trials Enrolling Older Adults Only and Those including Adults: A Meta-Epidemiological Study
Source: PLoS One. 2013 May 28;8(5):e63677. doi: 10.1371/journal.pone.0063677 (PMC3665786; doi:10.1371/journal.pone.0063677)
Supplement: Appendix S1 — Annexes. (DOCX) [file pone.0063677.s006.docx]

***Comparison of treatment effect estimates for pharmacological randomized controlled trials enrolling older adults only and other: a meta-epidemiological study***

***ANNEXES***

***APPENDIX A) SELECTION PROCESS***

**APPENDIX B) DESCRIPTION OF INCLUDED META-ANALYSES**

**APPENDIX C) OR CALCULATION IN EACH META-ANALYSIS FOR elderly-RCTs AND adults-RCTS SEPARATELY**

**APPENDIX D) ROR PER META-ANALYSES FROM FIXED EFFECTS AND FROM A META-REGRESSION MODEL**

**APPENDIX E) EXPLORATION OF HETEROGENEITY**

**APPENDIX F) SENSITIVITY ANALYSES BY AGE GROUP**

**APPENDIX G) DATA EXTRACTION FORM**

***APPENDIX A) SELECTION PROCESS***

| ***Definition of randomized controlled trial specifically including older adults (elderly- RCT)*** | 1. Randomised 2. elderly-RCTs: 3. Explicitly mentioned by authors 4. Age as inclusion criteria: ≥60 years 5. Range of age patients specified: min≥60 years 6. Mean age minus 2 SD ≥ 60 years 7. Mean age (or median ag) ≥ 75 years |
| --- | --- |
| ***Inclusion criteria of meta-analysis*** | 1. **Cochrane** systematic review with meta-analysis 2. All medical domains 3. Therapeutic intervention (pharmacologic or nonpharmacologic) 4. At least 3 RCTs, including 1 elderly-RCT (as defined above) 5. Published data |
| ***Exclusion criteria*** | 1. Nontherapeutic interventions (economic, public health organization, primary prevention) 2. Meta-analyses of individual patient data 3. No clearly elderly-RCTs or only elderly- RCTs 4. Fewer than 3 RCTs 5. Subgroups analysis based on age, without elderly-RCTs 6. Network meta-analysis |

***APPENDIX A) SELECTION PROCESS (continued)***

| ***Sélection process*** | 1. Selection on title: Exclusion if    1. Nontherapeutic intervention (eg, speed cameras for the prevention of road traffic injuries and deaths)    2. Clearly not elderly destination (eg, efficacy and safety of cesarean delivery for prevention of mother-to-child transmission of HIV-1)    3. Elderly specific interventions (eg, interventions to increase influenza vaccination rates of people ≥ 60 years in the community) 2. Selection on abstract. Exclusion if    1. individual patient data    2. No meta-analysis    3. Fewer than 3 RCTs included 3. Selection on full text. Exclusion if:    1. No elderly- RCTs    2. Only elderly- RCTs    3. <3 RCTs per meta-analysis |
| --- | --- |

**APPENDIX B) DESCRIPTION OF INCLUDED META-ANALYSES**

| Review | Medical domain | Intervention | Control | Selected outcome | OR | I² | elderly- RCTs | Mixed | older excluding | Child | Unclear |
| --- | --- | --- | --- | --- | --- | --- | --- | --- | --- | --- | --- |
| CD000039* | acute stroke | ACE inhibitors (per os) | Placebo or nothing | Case fatality  (within 1 month) | 1.09 (0.39-3.05) | 0% | 2 | 3 | 0 | 0 | 3 |
| CD000064 | Acute ischemic stroke | Corticosteroids | Placebo | all death | 0.88 (0.51-1.49) | 23.8% | 1 | 4 | 0 | 0 | 3 |
| CD000096* | Acute stroke | intravenous glycerol | No glycerol | Death within the scheduled treatment period | 0.81 (0.56-1.16) | 19.8% | 2 | 7 | 0 | 0 | 1 |
| CD000248 | Non-embolic ischemic stroke and transcient ischemic attack | Anticoagulant | Placebo or nothing | Death of any cause during follow-up | 0.95 (0.73-1.24) | 0% | 1 | 5 | 1 | 0 | 3 |
| CD000419 | Stroke | Piracetam | Placebo or nothing | Death at approximately  1 month | 1.34 (0.96-1.86) | 0.2% | 1 | 2 | 0 | 0 | 0 |
| CD000424* | Aphasia following strke | Piracetam compared | Placebo | Nb of patients with aphasia not improved at the end of the study | 0.27 (0.08-0.88) | 71% | 1 | 3 | 0 | 0 | 0 |
| CD001100* | VTE | Fixed dose subcutaneous LMWH | Adjusted dose unfractionated heparin | Nb of recurrent VTE during initial treatment | 0.52 (0.20-1.4) | 0% | 1 | 2 | 0 | 0 | 1 |
| CD001359 | Schizophrenia | Olanzapine | Atypical antipsychotic | Death | 0.70 (0.30-1.61) | 0% | 2 | 0 | 1 | 0 | 0 |
| CD001390 | Chronic obstructive pulmonary disease | Pneumococcal vaccine | Placebo or nothing | Nb of pneumonia | 0.69 (0.37-1.29) | 51.6% | 2 | 2 | 1 | 0 | 0 |
| CD001447 | Amyotrophic lateral sclerosis | Riluzole 100 mg | Placebo | Mortality  at 12 months | 0.65 (0.46-0.92) | 21.2% | 1 | 0 | 1 | 0 | 1 |
| CD001841 | Hypertension | Low-dose thiazide | Placebo | Total mortality | 0.95 (0.81-1.1) | 25.4% | 1 | 1 | 1 | 0 | 2 |
| CD001884 | Peri-operative allogenic blood transfusion | Desmopressin | Placebo or nothing | Nb exposed to allogenic blood | 1.09 (0.84-1.42) | 10.7% | 1 | 16 | 0 | 0 | 2 |
| CD001886* | Peri-operative allogenic blood transfusion | Tranexamic Acid | Placebo or nothing | Nb exposed to allogenic blood | 0.39 (0.32-0.47) | 41.1% | 5 | 53 | 2 | 0 | 4 |
| CD001944* | Schizophrenia | Thioridazine | Atypical Antipsychotic | Global state: not improved or worse | 0.98 (0.52-1.85) | 0% | 1 | 0 | 2 | 0 | 0 |

ACE: angiotensin-converting enzyme, VTE: venous thrombo-embolic, LMWH: low-molecular-weight heparins; * the magnitude of the odds ratio (OR) in elderly- and adults- RCTs differed by at least double in these systematic reviews

**Appendix B (continued)**

| Review | Medical domain | Intervention | Control | Selected outcome | OR | I² | elderly- RCTs | Mixed | older excluding | Child | Unclear |
| --- | --- | --- | --- | --- | --- | --- | --- | --- | --- | --- | --- |
| CD002001* | Symptomatic VTE | LMWH | VKA | Incidence of reccurent VTE during 3 months of allocated treatment | 0.82 (0.39-1.74) | 37.6% | 1 | 0 | 0 | 0 | 6 |
| CD002003 | Hypertension | β-blocker | Placebo or nothing | Total mortality | 0.99 (0.87-1.12) | 0% | 2 | 0 | 2 | 0 | 0 |
| CD002090 | Recovery after stroke | Amphetamine | Placebo | Dead or dependant at the end of follow-up | 1.46 (0.63-3.37) | 0% | 1 | 1 | 1 | 0 | 0 |
| CD002146 | Hypertension | Hypertension Treatment | Placebo or Usual Care | Fatal cerebro-vascular events | 0.78 (0.58-1.05) | 0% | 2 | 5 | 0 | 0 | 4 |
| CD002747* | Metastatic breast cancer | Endocrine therapy | Chemotherapy | Tumor response rate | 0.79 (0.39-1.62) | 75% | 2 | 4 | 1 | 0 | 0 |
| CD002755 | Acute myocardial infarction | Magnesium | Placebo | Mortality by time of admission < 6 h | 0.88 (0.72-1.08) | 57.4% | 1 | 5 | 1 | 0 | 1 |
| CD002839 | Acute stroke | Drug | Placebo or nothing | Early death | 1.16 (0.98-1.37) | 0% | 4 | 26 | 0 | 0 | 0 |
| CD003178 | Congestive heart failure | Continuous Infusion of loop diuretics | Bolus Injection of loop diuretics | Clinically relevant changes in blood chemistry hypokaliema and hypomagnemesia | 0.67 (0.09-4.74) | 43.9% | 1 | 2 | 0 | 0 | 0 |
| CD003189 | Malignant lymphoma | G-CSF/GM-CSF | Placebo or no prophylaxis | overall survival | 0.93 (0.78-1.11) | 0% | 4 | 3 | 4 | 0 | 0 |
| CD003197 | Depression | Low dosage TCA | Placebo | Acceptability of treatment  (as measured by leaving study early for any reason) | 0.88 (0.70-1.11) | 0% | 5 | 1 | 11 | 0 | 10 |
| CD003229 | Venous insufficiency | Rutosides | Placebo | Oedema in the lower legs | 0.34 (0.21-0.55) | 30.1% | 1 | 3 | 0 | 0 | 3 |
| CD003344 | Sepsis | β-lactam antibiotic monotherapy | Different β-lactam-aminoglycoside antibiotic combination | All cause fatality | 0.8 (0.62-1.04) | 19.7% | 1 | 0 | 0 | 0 | 21 |
| CD003348 | Post-operative pain | Patient controlled opioid analgesia | Conventional opioid analgesia | Nb of patients in arm satisfied with therapy | 2.97 (1.62-5.41) | 42% | 2 | 0 | 0 | 0 | 10 |
| CD003654 | Hypertension | CCBs | Diuretic or blocker | All cause mortality | 1.03 (0.94-1.13) | 0% | 1 | 1 | 0 | 0 | 1 |

ACE: angiotensin-converting enzyme, VTE: venous thrombo-embolic, LMWH: low-molecular-weight heparins; * the magnitude of the OR in elderly- and adults- RCTs differed by at least double in these systematic reviews

**Appendix B (continued)**

| Review | Medical domain | Intervention | Control | Selected outcome | OR | I² | elderly- RCTs | Mixed | older excluding | Child | Unclear |
| --- | --- | --- | --- | --- | --- | --- | --- | --- | --- | --- | --- |
| CD003781* | Overactive bladder syndrom | Anticholinergics | Placebo | Patient perception of cure improvement | 0.47 (0.34-0.65) | 60.6% | 3 | 2 | 0 | 0 | 3 |
| CD003838 | Heart failure | Diuretic | Placebo | Mortality | 0.24 (0.07-0.84) | 0% | 2 | 1 | 0 | 0 | 0 |
| CD004125* | Post-operative nausea and vomiting | Clonidine | Placebo | Rescue anti-emetic | 1.1 (0.65-1.85) | 0% | 1 | 0 | 2 | 1 | 3 |
| CD004171* | Allogenic blood transfusion | Fibrin sealant | Usual care | Nb exposed to allogenic transfusion | 0.51 (0.33-0.79) | 35.3% | 1 | 13 | 0 | 0 | 4 |
| CD004185 | Depression | Fluoxetine | Amitriptyline | Failure to respond | 1.02 (0.75-1.4) | 0% | 2 | 0 | 4 | 0 | 3 |
| CD004186 | Depression | trazodone | Amitriptyline | Responders | 1.02 (0.71-1.47) | 25.2% | 1 | 3 | 0 | 0 | 4 |
| CD004196 | Uninstable angina pectoris | Puerarin and routine | Routine | Reduction in attacks of acute angina attacks | 0.3 (0.19-0.46) | 37.6% | 2 | 12 | 1 | 0 | 1 |
| CD004310* | Anorexia-cachexia syndrome | Megestrol acetate | Placebo | Weight gain | 0.36 (0.25-0.51) | 11.7% | 1 | 6 | 0 | 1 | 2 |
| CD004386 | Afebrile neutropenic following chemotherapy | Quinolone | Placebo/ no intervention | Infection related mortality | 0.59 (0.34-1.03) | 10.5% | 1 | 0 | 0 | 0 | 11 |
| CD004418 | Community acquired pneumonia | Quinolone (atypical arm) | Quinolone (typical arm) | Mortality per-antibiotic treatment | 1.01 (0.68-1.49) | 0% | 3 | 7 | 1 | 0 | 2 |
| CD005237 | Schizophrenia | Risperidone | Olanzapine | Global effect | 0.81 (0.5-1.30) | 38.3% | 1 | 0 | 2 | 0 | 0 |
| CD005259 | Thrombo-embolism and knee arthroscopy | Interventions for preventing VTE  (all LMWH) | No treatment | Nb of participants with thrombotic events | 0.14 (0.04-0.50) | 0% | 1 | 2 | 0 | 0 | 0 |
| CD005429 | Overactive bladder syndrom | Tolterodine | Oxybutynin | Cure or improvement | 0.97 (0.78-1.21) | 0% | 1 | 0 | 0 | 0 | 4 |
| CD005454* | Neuropathic pain | Amitriptyline | Placebo | Nb of patients with moderate pain relief or better | 0.19 (0.08-0.46) | 75.9% | 1 | 3 | 3 | 0 | 3 |
| CD006114* | Depression | Fluvoxamine | TCAs | Response at acute phase | 1.03 (0.77-1.37) | 6.1% | 2 | 1 | 13 | 0 | 0 |

TCAs: tricyclic antidepressants, VTE: venous thrombo-embolic; * the magnitude of the OR in elderly- and adults- RCTs differed by at least double in these systematic reviews

**Appendix B (continued)**

| cdsr | medical.domain | | | Intervention | | Control | selected.outcome | | OR | | I² | | Eld RCTs | | Mixt | older excluding | child | | Unclear | |
| --- | --- | --- | --- | --- | --- | --- | --- | --- | --- | --- | --- | --- | --- | --- | --- | --- | --- | --- | --- | --- |
| CD006117 | | Depression | Sertraline | | Amitriptyline | | Failure to respond at endpoint (6-12 weeks) | 1.23 (0.99-1.52) | | 0% | | 1 | | 1 | | 1 | | 0 | | 4 |
| CD006186 | Non valvular atrial fibrilation | | | Oral anticoagulant | | Antiplatelet | All strokes | | 0.68 (0.54-0.85) | | 0% | | 6 | | 2 | 0 | 0 | | 0 | |
| CD006369 | Schizophrenia | | | Paliperidone | | Placebo | Leaving the study | | 0.58 (0.37-0.93) | | 74.1% | | 1 | | 0 | 2 | 0 | | 4 | |
| CD006529 | Depression | | | Milnacipran | | Imipramine | Response at acute phase  (6-12 weeks) | | 0.96 (0.65-1.41) | | 0% | | 1 | | 0 | 2 | 0 | | 0 | |
| CD006532 | Depression | | | Escitalopram | | Fluoxetine | Failure to respond at endpoint (6-12 weeks) | | 0.81 (0.60-1.1) | | 0% | | 1 | | 1 | 1 | 0 | | 0 | |
| CD006626* | Schizophrenia | | | Risperidone | | Amisulpride | Leaving the study early | | 0.92 (0.56-1.50) | | 0% | | 1 | | 0 | 3 | 0 | | 0 | |
| CD006654 | Schizophrenia | | | Olanzapine** | | Risperidone** | No clinically significant response | | 1.12 (0.86-1.46) | | 14.8% | | 1 | | 0 | 4 | 1 | | 1 | |
| CD006743 | Acute cardio-vascular event | | | CCB short-term treatment | | Placebo | All cause mortality at 10 days | | 1.00 (0.70-1.44) | | 0% | | 1 | | 13 | 0 | 0 | | 1 | |
| CD007503* | Depression | | | Antidepressants | | Placebo | Response to treatment  (4-5 weeks) | | 0.44 (0.22-0.86) | | 59.9% | | 1 | | 2 | 0 | 0 | | 4 | |
| CD007938 | Neuropathic pain and fibromyalgia | | | Gabapentin | | Placebo | Pain reduction (at least 50pct over baseline) | | 0.47 (0.28-0.79) | | 50.8% | | 1 | | 1 | 1 | 0 | | 0 | |
| CD008120* | Anxiety disorders | | | Quetiapine monotherapy | | Placebo | Response | | 0.45 (0.22-0.91) | | 93.2% | | 1 | | 0 | 3 | 0 | | 0 | |
| CD008238 | Haematology | | | Colony-stimulating factors | | Placebo or nothing | All cause mortality  at 30 days | | 0.97 (0.77-1.21) | | 0% | | 1 | | 5 | 1 | 1 | | 3 | |

CCB: calcium channel blockers, ** intervention and control were reversed in the analyses (Olanzapine was before to Risperidone, then we considered olanzapine as control treatment) * the magnitude of the OR in elderly- and adults- RCTs differed by at least double in these systematic reviews

**APPENDIX C) OR CALCULATION IN EACH META-ANALYSIS FOR elderly-RCTs AND adults- RCTS SEPARATELY**

| CDSR | Nb eld RCTs | OR eld | SeOR | I² % | τ² | Nb non-eld  RCTs | OR non-eld | Se OR | I² | τ² |
| --- | --- | --- | --- | --- | --- | --- | --- | --- | --- | --- |
| CD000064 | 1 | 0.93 | 0.44 |  |  | 7 | 0.87 | 0.35 | 34 | 0.28 |
| CD000039 | 2 | 1.98 | 0.96 | 0 | 0 | 6 | 0.84 | 0.63 | 0 | 0 |
| CD000248 | 1 | 0.58 | 0.67 |  |  | 9 | 0.97 | 0.14 | 0 | 0 |
| CD000419 | 1 | 1.08 | 0.86 |  |  | 2 | 0.79 | 0.98 | 49 | 0.13 |
| CD000424 | 1 | 0.09 | 0.63 |  |  | 3 | 0.52 | 0.39 | 24 | 16 |
| CD001100 | 1 | 0.24 | 1.13 |  |  | 3 | 0.63 | 0.56 | 0 | 0 |
| CD001359 | 2 | 0.96 | 1.16 | 0 | 0 | 1 | 0.66 | 0.46 |  |  |
| CD001390 | 2 | 0.65 | 0.50 | 82 | 0.41 | 3 | 0.75 | 0.53 | 29 | 0.24 |
| CD001447 | 1 | 0.98 | 0.32 |  |  | 2 | 0.57 | 0.17 | 0 | 0 |
| CD001841 | 1 | 1.08 | 0.10 |  |  | 4 | 0.89 | 0.09 | 11 | 0 |
| CD001884 | 1 | 1.56 | 0.94 |  |  | 18 | 1.09 | 0.14 | 15 | 0.05 |
| CD001886 | 5 | 0.17 | 0.36 | 5 | 0.03 | 59 | 0.41 | 0.10 | 39 | 0.21 |
| CD001944 | 1 | 6.33 | 1.62 |  |  | 2 | 0.91 | 0.33 | 0 | 0 |
| CD002001 | 1 | 2.04 | 1.24 |  |  | 6 | 0.76 | 0.41 | 44 | 0.42 |
| CD002003 | 2 | 1.05 | 0.09 | 0 | 0 | 2 | 0.93 | 0.09 | 0 | 0 |
| CD002090 | 1 | 2.17 | 0.67 |  |  | 2 | 1.11 | 0.56 | 0 | 0 |
| CD002146 | 2 | 1.01 | 0.28 | 0 | 0 | 9 | 0.71 | 0.18 | 0 | 0 |
| CD002747 | 2 | 1.60 | 0.27 | 0 | 0 | 5 | 0.51 | 0.34 | 52 | 0.28 |
| CD002755 | 1 | 1.00 | 0.07 |  |  | 7 | 0.69 | 0.21 | 63 | 0.11 |
| CD002839 | 4 | 0.75 | 0.54 | 0 | 0 | 26 | 1.17 | 0.09 | 0 | 0 |
| CD003178 | 1 | 1.00 | 1.13 |  |  | 2 | 0.59 | 1.81 | 69 | 4.60 |
| CD003189 | 4 | 0.89 | 0.10 | 0 | 0 | 7 | 1.15 | 0.22 | 0 | 0 |
| CD003197 | 5 | 0.90 | 0.29 | 0 | 0 | 22 | 0.88 | 0.13 | 0 | 0 |
| CD003229 | 1 | 0.56 | 0.41 |  |  | 6 | 0.30 | 0.28 | 31 | 0.14 |
| CD003344 | 1 | 0.75 | 0.80 |  |  | 21 | 0.80 | 0.14 | 23 | 0.08 |
| CD003348 | 2 | 3.11 | 0.44 | 0 | 0 | 10 | 3.04 | 0.39 | 53 | 0.73 |
| CD003654 | 1 | 0.99 | 0.08 |  |  | 2 | 1.05 | 0.06 | 0 | 0 |
| CD003781 | 3 | 0.18 | 0.33 | 0 | 0 | 5 | 0.56 | 0.10 | 17 | 0.01 |
| CD003838 | 2 | 0.26 | 0.69 | 0 | 0 | 1 | 0.16 | 1.58 |  |  |
| CD004125 | 1 | 0.46 | 1.28 |  |  | 6 | 1.15 | 0.29 | 8 | 0.04 |
| CD004171 | 1 | 0.10 | 1.59 |  |  | 17 | 0.52 | 0.23 | 36 | 0.25 |
| CD004185 | 2 | 0.58 | 0.79 | 73 | 0.92 | 7 | 1.11 | 0.18 | 0 | 0 |
| CD004186 | 1 | 1.27 | 0.43 |  |  | 7 | 0.97 | 0.22 | 34 | 0.11 |
| CD004196 | 2 | 0.25 | 0.59 | 0 | 0 | 14 | 0.30 | 0.25 | 45 | 0.37 |
| CD004310 | 1 | 0.81 | 0.57 |  |  | 9 | 0.33 | 0.18 | 0 | 0 |
| CD004386 | 1 | 0.94 | 1.42 |  |  | 11 | 0.57 | 0.31 | 18 | 0.18 |
| CD004418 | 3 | 1.59 | 0.48 | 0 | 0 | 10 | 0.91 | 0.22 | 0 | 0 |
| CD005237 | 1 | 0.79 | 0.32 |  |  | 2 | 0.71 | 0.50 | 68 | 0.35 |
| CD005259 | 1 | 0.19 | 1.11 |  |  | 2 | 0.13 | 0.77 | 0 | 0 |
| CD005429 | 1 | 0.85 | 0.21 |  |  | 4 | 1.03 | 0.13 | 0 | 0 |
| CD005454 | 1 | 0.34 | 0.57 |  |  | 9 | 0.17 | 0.51 | 79 | 1.66 |
| CD006114 | 2 | 0.53 | 0.72 | 59 | 0.61 | 14 | 1.09 | 0.15 | 0 | 0 |
| CD006117 | 1 | 0.92 | 0.27 |  |  | 6 | 1.30 | 0.12 | 0 | 0 |
| CD006186 | 6 | 0.67 | 0.13 | 0 | 0 | 2 | 0.76 | 0.35 | 18 | 0.04 |
| CD006369 | 1 | 0.41 | 0.47 |  |  | 6 | 0.62 | 0.27 | 78 | 0.30 |
| CD006529 | 1 | 0.96 | 0.27 |  |  | 2 | 0.96 | 0.29 | 0 | 0 |
| CD006532 | 1 | 0.71 | 0.22 |  |  | 2 | 0.91 | 0.21 | 0 | 0 |
| CD006626 | 1 | 2.09 | 1.46 |  |  | 3 | 0.90 | 0.25 | 0 | 0 |
| CD006654 | 1 | 1.09 | 0.30 |  |  | 6 | 1.13 | 0.17 | 29 | 0.05 |
| CD006743 | 1 | 1.00 | 0.87 |  |  | 14 | 1.00 | 0.19 | 0 | 0 |
| CD007938 | 1 | 0.34 | 0.31 |  |  | 2 | 0.57 | 0.30 | 34 | 0.07 |
| CD008120 | 1 | 0.15 | 0.21 |  |  | 3 | 0.67 | 0.10 | 0 | 0 |
| CD008238 | 1 | 0.69 | 0.40 |  |  | 10 | 1.00 | 0.12 | 0 | 0 |
| CD007503 | 1 | 1.23 | 0.51 |  |  | 6 | 0.36 | 0.35 | 52 | 0.37 |
| CD000096 | 2 | 0.39 | 0.33 | 0 | 0 | 8 | 0.97 | 0.18 | 0 | 0 |

OR<1 favors experimental treatment

**APPENDIX E) EXPLORATION OF HETEROENEITY**

**Exploration of heterogeneity.** Each point on the scatterplot represents a meta-analysis. The x-axis represents the contribution of the meta-analysis to the overall heterogeneity across the ratio of odds ratios (RORs). The y-axis represents the contribution of the meta-analysis to the overall combined ROR. Three meta-analyses accounted for half of the heterogeneity (50.0% of the overall heterogeneity) and had a strong inﬂuence on the combined ROR (64.9% of the sum of the inﬂuences).

| Exclusion of | No. of MAs included in analysis | Combined ROR (95%CI) |
| --- | --- | --- |
| Main analysis | 55 | 0.91 (0.77-1.08)  I²=51%, Tau²=0.137 |
| The most heterogenous MA (CD008120) | 54 | 0.97 (0.85-1.11)  I²=25%, Tau²=0.04 |
| The 3 most heterogenous MA excluded (CD008120, CD003781, CD002747) | 52 | 0.99 (0.90- 1.10)  I²=2%, Tau²=0.0035 |

MA, meta-analysis

| Sub-group |  | No. of MAs included in analysis | Combined ROR (95%CI) |
| --- | --- | --- | --- |
| Type of control Arm | Pacebo or usual care | 35 | 0.87 (0.68-1.11) |
|  | Active drug | 20 | 0.95 (0.82-1.10) |
| Type of outcome | Mortality | 19 | 1.11 (0.90-1.36) |
|  | Other outcome | 36 | 0.86 (0.66-1.13) |
| Type of outcome | Primary outcome of the included MA | 36 | 0.88 (0.70-1.10) |
|  | Secondary outcome of the included MA | 19 | 0.91 (0.77-1.08) |

**APPENDIX F) SENSITIVITY ANALYSES BY AGE-GROUP RCT**

|  | elderly- RCTs  compared to | No. of MAs included in analysis | Combined ROR (95%CI) | HMM |
| --- | --- | --- | --- | --- |
| Main Analysis elderly-RCTs vs adults-RCTs | | 55 | 0.91 (0.77-1.08) | 0.87(0.75-1.04) |
| Unclear separately considered | |  |  |  |
|  | mixed-adults”RCTs | 38 | 0.93 (0.76-1.15) | 0.95 (0.83-1.09) |
|  | older-excluding RCTs | 29 | 0.86 (0.63-1.17) | 0.88 (0.70-1.09) |
|  | Unclear | 34 | 0.96 (0.82-1.12) | 0.96 (0.82-1.12) |
| Unclear considered in one and in the other group successively | | |  |  |
|  | mixed-adults & unclear | 48 | 0.95 (0.80-1.11) | 0.92 (0.81-1.05) |
|  | older-excluding RCTs & unclear age-group RCTs | 48 | 0.93 (0.78-1.12) | 0.92 (0.80-1.06) |

HMM: Hierarchic mixed model

**APPENDIX G) DATA EXTRACTION FORM**

DATA EXTRACTION FORM: META-ANALYSIS PT & binary

# PART 1:

| **Meta-Analysis ID**: ………………….. | **Reviewer ID:** |
| --- | --- |
| **Date of publication (year):**…………. | **Cochran Group:** ……………….……………… |

# ITEMS PICO

**Pathology:** ………………………………..……………………………………….….….

**Medical domain:** ………………………………………………………………….…….

**Experimental treatment studied:** **🞏PT:** ……………………………..

**Control treatment studied:** **🞏PT:** ………………………………

**🞏Placebo 🞏No treatment**

# Assessment of quality

**Cochrane ROB Tool : 🞏 yes 🞏no Nb of evaluated ROB items : ………….**

# From TABLE 1 : Number of included studies:…………………………………..

# Elderly specific RCTs:

| Name of elderly RCT | Age characteristics reported | Name of elderly RCT | Age characteristics reported |
| --- | --- | --- | --- |
|  |  |  |  |
|  |  |  |  |
|  |  |  |  |
|  |  |  |  |
|  |  |  |  |
|  |  |  |  |

# PART 2: From Results section or SR/MA, Choice of the outcome to extract:

**🞏 CASE 1: 1 eligible MA is the main efficacy outcome of the SR/MA**

**🞏 CASE 2: Eligible MA is(are) not the main efficacy outcome(s) of the SR/MA**

**If case 2, choose the first efficacy and binary outcome presented in SR/MA**

**Outcome studied: Comp …………….. Outcome……………….**

**…………………………………………………………………………..**

| **1/2/3/4/5**  **RCT** | **Study** | **Nb events exp arm** | **Nb patients arm** | **Nb events control arm** | **Nb patients control arm** |
| --- | --- | --- | --- | --- | --- |
|  |  |  |  |  |  |
|  |  |  |  |  |  |
|  |  |  |  |  |  |
|  |  |  |  |  |  |
|  |  |  |  |  |  |
|  |  |  |  |  |  |
|  |  |  |  |  |  |
|  |  |  |  |  |  |
|  |  |  |  |  |  |
|  |  |  |  |  |  |
|  |  |  |  |  |  |
|  |  |  |  |  |  |
|  |  |  |  |  |  |
|  |  |  |  |  |  |
|  |  |  |  |  |  |
|  |  |  |  |  |  |

**Eld RCT: 1 ; Mixte RCT: 2 ; Mid-aged : 3 ; Unclear: 4 ; Unspecif: 5**
